# Characteristics, Management, and Outcomes of Inferior Scapula Angle Fractures: A Systematic Review of the Literature

**DOI:** 10.7759/cureus.27192

**Published:** 2022-07-24

**Authors:** Vasileios K Mousafeiris, Nektaria Kalyva, Nikolaos Rigopoulos, Francesk Mulita, Konstantinos Mousafiris

**Affiliations:** 1 Orthopedics and Traumatology, General Hospital of Patras "Agios Andreas", Patras, GRC; 2 Pediatrics, University Hospital of Patras, Patras, GRC; 3 Pediatric Orthopedics, University Hospital of Larissa, Larissa, GRC; 4 Surgery, University Hospital of Patras, Patras, GRC; 5 Orthopedics and Traumatology, University Hospital of Patras, Patras, GRC

**Keywords:** operative, conservative, management, displacement, inferior scapula angle fracture, inferior angle, scapula

## Abstract

Fractures of the inferior angle of the scapula represent a rare entity, with few cases published in the literature to date. Their optimal management is still unclear. A systematic literature search was conducted in PubMed and Google Scholar of reports published between 1977 and 2022. Inclusion criteria were cases presenting inferior scapula angle fractures (ISAF) and reporting management and outcomes. Extracted data included patient demographics (age, sex), mechanism of injury, associated injuries, management, procedures performed, and outcomes. Seventeen studies (22 cases; 19 males and three females) were included. The mean age was 33 years (15 adult and seven pediatric cases). High energy injuries were the most common mechanism of injury (77%). Displacement of the fragment was reported in 64%. The most common initial treatment was conservative (77%); of these cases, more than half failed initial treatment (53%). Of the displaced ISAF cases, 89% failed conservative treatment, contrary to 83% of the nondisplaced cases that were successfully treated conservatively. Surgery yielded 100% success both as initial treatment and after failed conservative management. Displaced fractures should be approached surgically, while conservative management should be reserved for nondisplaced fractures.

## Introduction and background

Fractures of the scapula are relatively rare and represent about only 3-5% of the total shoulder area fractures [1]. The majority are considered high-energy injuries; however, avulsion fractures after low-energy injuries have been described [2]. Inferior scapula angle fractures (ISAF) are more uncommon with very few cases published to date. They have been reported either after high energy trauma or after low energy or indirect trauma. Both conservative and surgical management has been described, but it is unclear whether the suggestions made by recent studies [3] are still valid. The purpose of this review is to summarize the available evidence regarding ISAF, including management and outcomes.

## Review

Materials and methods

A systematic literature review was conducted to identify the available literature. Literature published between 1977 and 2022 in MEDLINE (through PubMed) and the first 200 articles in the Google Scholar electronic database were included. Specific search strings were formulated in PubMed using the following keywords and/or Medical Subject Headings (MeSH) terms: “(scapula OR shoulder blade) AND (angle OR border OR part) AND (inferior OR low OR lower) AND (fractur* OR avulsion OR avulsed OR detachment OR detached OR injur* OR trauma OR traumatic OR winging)”. The keywords "scapula", "inferior angle" and "fracture" were used in Google Scholar. This study was conducted according to the 2009 Preferred Reporting Items for Systematic Review and Meta-analysis (PRISMA) statement [4].

Inclusion criteria were studies reporting on ISAF mechanism of injury (MOI), management, and outcomes. Exclusion criteria were biomechanical studies, animal studies, review articles, post-mortem studies, editorials, comments, opinions, letters to the editor, published abstracts, and errata (unless they provide original data). The reference lists of the included studies were cross-checked to identify additional relevant studies.

Data extracted from the eligible studies included: patient demographics (age, sex), MOI, associated injuries, management, procedures performed, and outcomes. All data was inserted into an electronic database for subsequent analysis. At the same time, we include our case of ISAF, to further enrich the scientific literature.

Supplement original case of ISAF after extreme shoulder adduction

A 42-year-old male was admitted to our Emergency Room (ER) for pain in the posterior thorax on the left side, on the area overlying the lower part of the scapula. The patient recalled no trauma but reported that while trying to take off a very narrow shirt, the shirt was torn on the right side at the level of the lower scapula. The patient, instead of unbuttoning the shirt, tried to tear it completely from the top to bottom to remove it that way. Immediately after, the patient heard a "crack" sound and felt pain on the posterolateral thorax on the left side.

**Figure 1:**
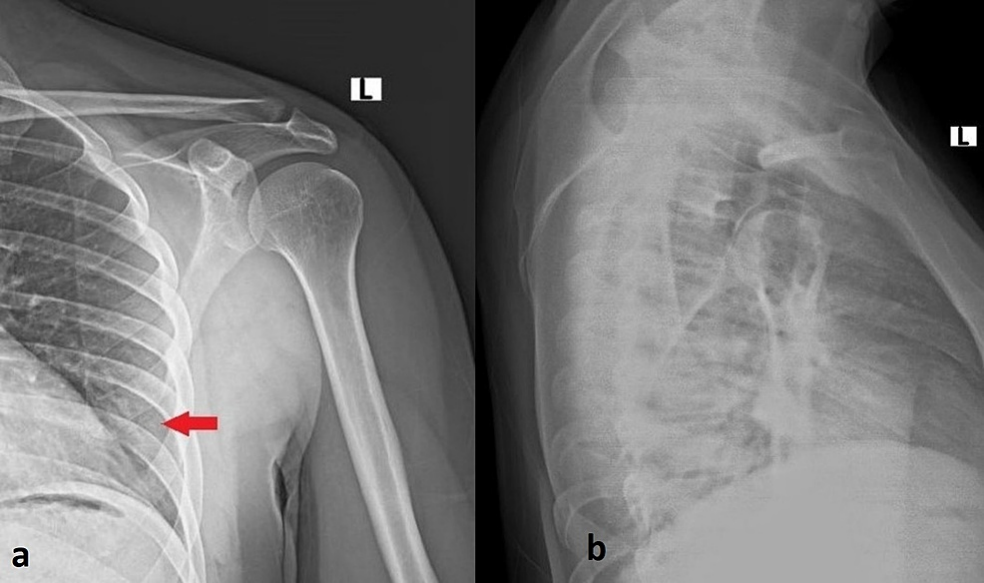
Anteroposterior (a) and transthoracic (b) shoulder x-rays did not reveal any fracture Red arrow depicts where the ISAF is located ISAF: inferior scapula angle fracture

During the physical exam, there was a mildly limited active range of motion (ROM) of the right shoulder (35 and 30 degrees deficit in the abduction and forward flexion, respectively). Passive ROM was, however, within normal limits. The entire left upper extremity was neurovascularly intact. The patient was also cleared by the surgical trauma team of our ER. Initial anteroposterior (AP) and transthoracic lateral x-ray imaging (Figure 1) of the left shoulder, scapula, and upper arm revealed no fracture.

**Figure 2:**
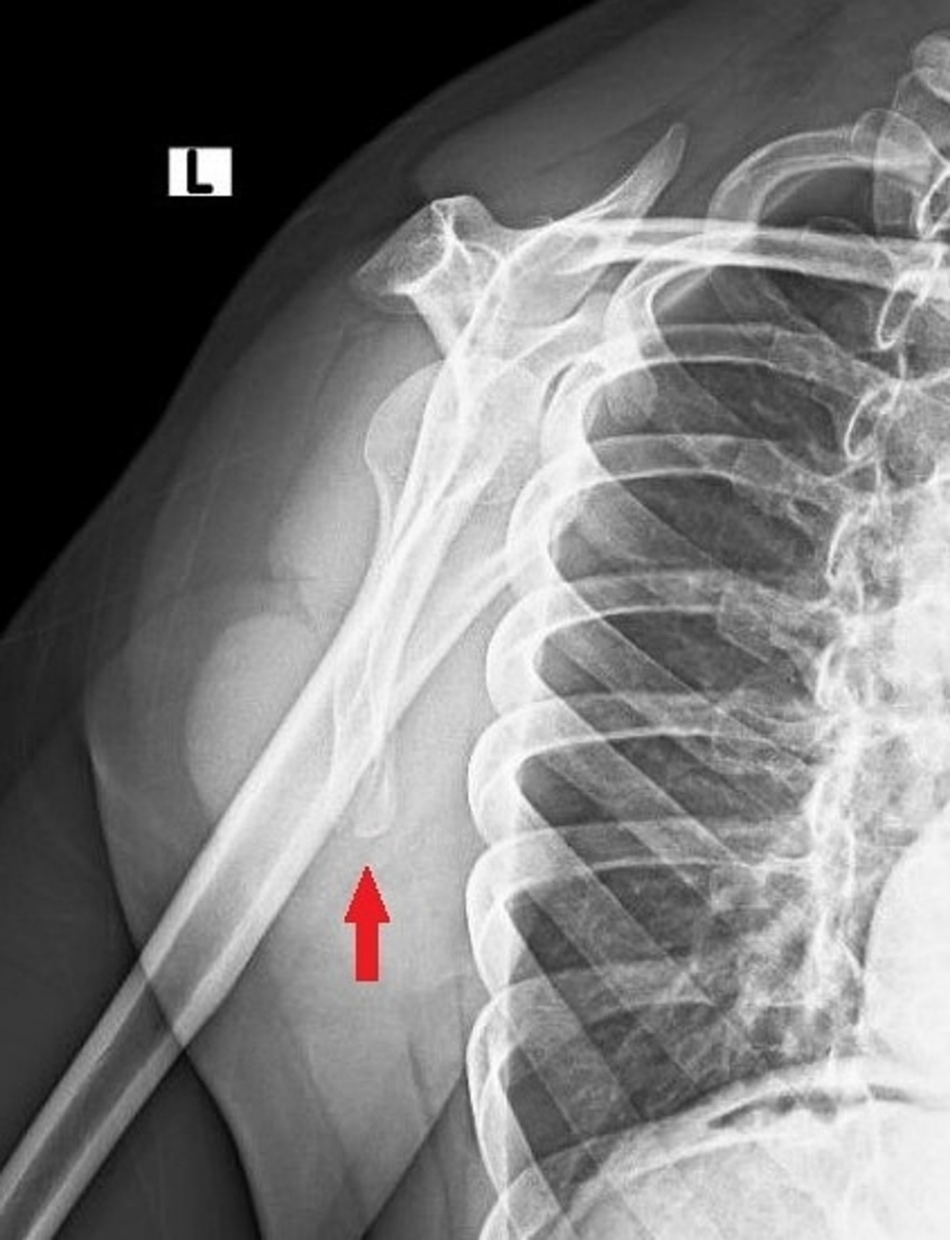
Lateral scapula x-ray revealed the ISAF (red arrow) ISAF: inferior scapula angle fracture

However, a lateral view of the left shoulder and scapula revealed a nondisplaced fracture of the inferior angle of the left scapula (Figure 2).

Shoulder functionality was also assessed with standardized scores. American Shoulder and Elbow Surgeons (ASES) score [5] was 26, Shoulder Pain and Disability Index (SPADI) score [6] was 78.5%, and Quick Disabilities of the Arm, Shoulder, and Hand (QuickDASH) score [7] was 75%.

**Figure 3:**
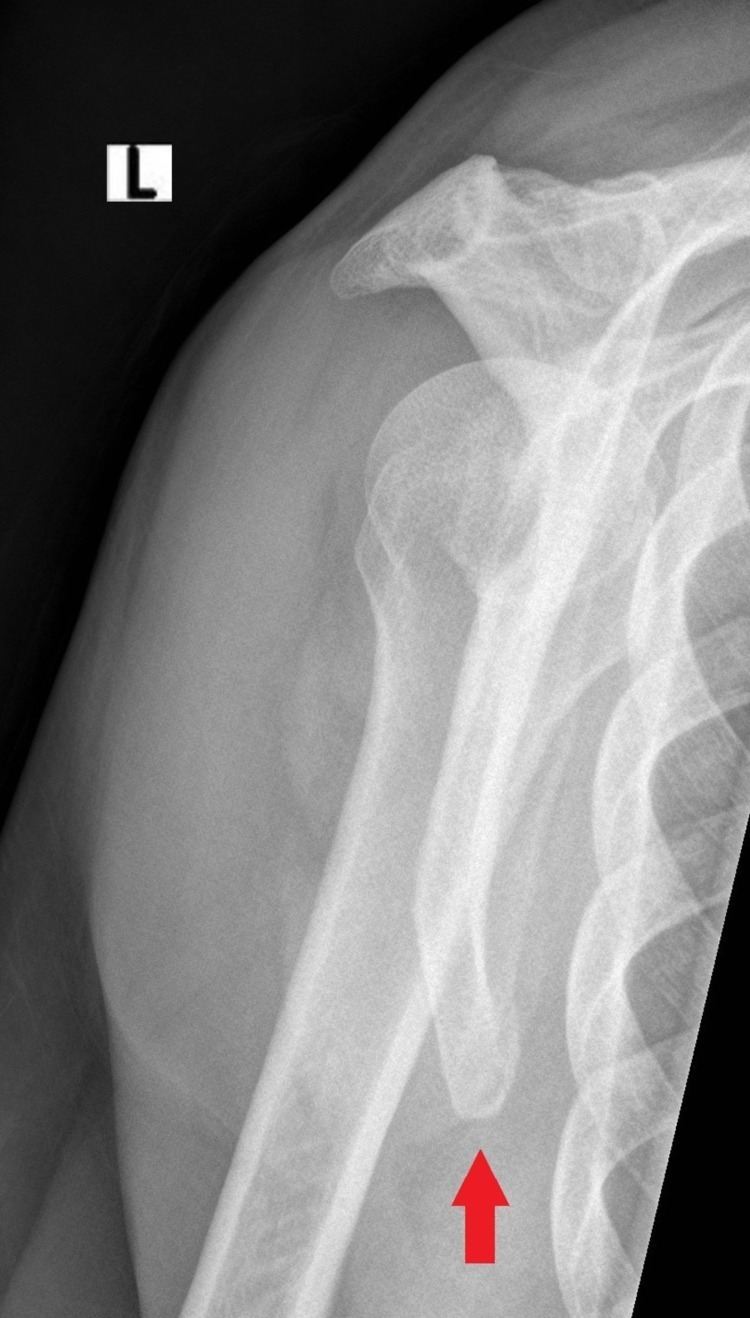
Lateral x-ray at 1.5-month follow-up revealed almost complete fracture healing (red arrow)

The patient was treated with a sling and instructions for pain management and was discharged home. On the 10-day follow-up, the patient reported improvement in pain and ROM. There was mild pain and slightly decreased active ROM but was able almost pain-free to perform passive ROM of the left shoulder. On the 1.5-month follow-up, the patient showed marked improvement in pain and passive and active ROM of the left shoulder. There was mild tenderness over the scapula area; however, the patient was able to initiate and maintain active abduction and forward flexion with minimal pain. ASES score was 77, SPADI score was 13.1%, and QuickDASH score was 20.5%. X-rays of the left shoulder showed fracture healing process (Figure 3).

**Figure 4:**
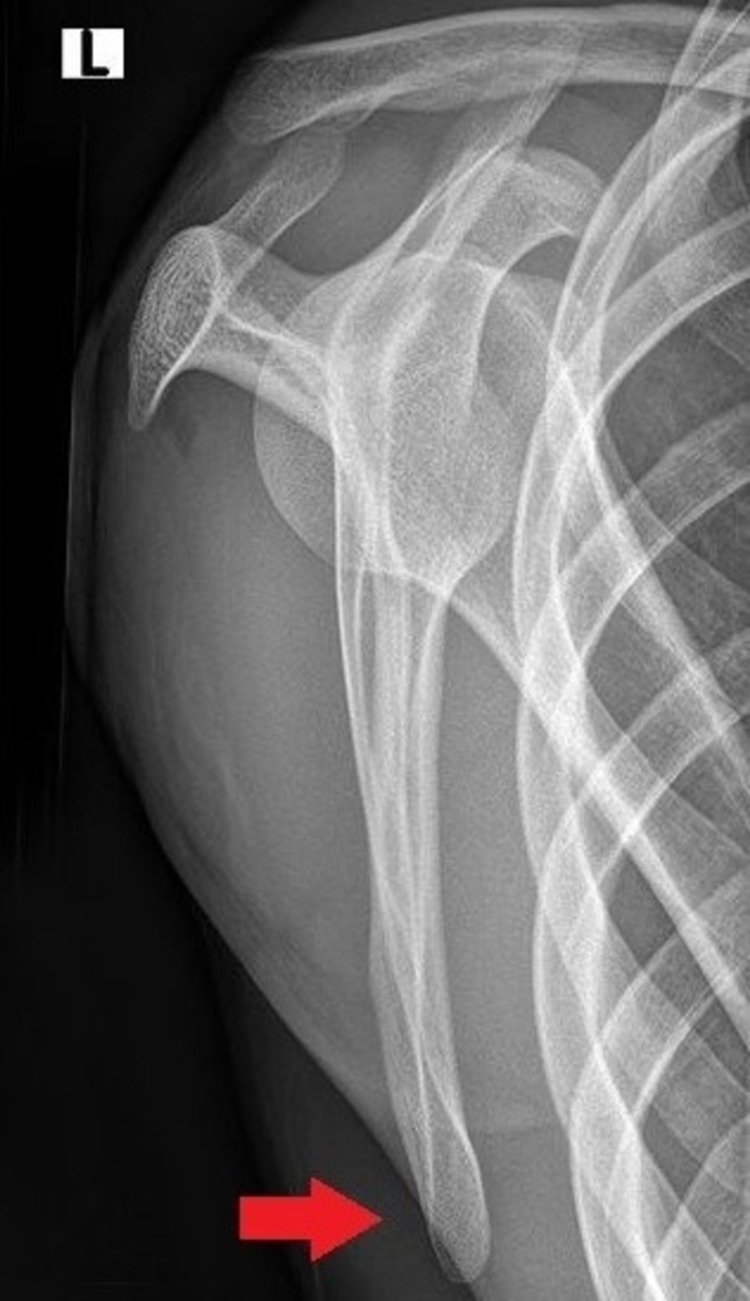
Lateral x-ray at three-month follow-up revealed complete fracture healing (red arrow)

On the three-month follow-up, the patient reported an excellent outcome with no pain at rest and with passive or active ROM. There was no tenderness, and the patient was able to initiate and maintain forward flexion and abduction of the left shoulder without any limitation. ASES score was 93, SPADI score was 3.1%, and QuickDASH score was 2.3%. X-rays showed complete healing of the fracture; therefore, the patient was discharged from our care (Figure 4).

Results

**Figure 5:**
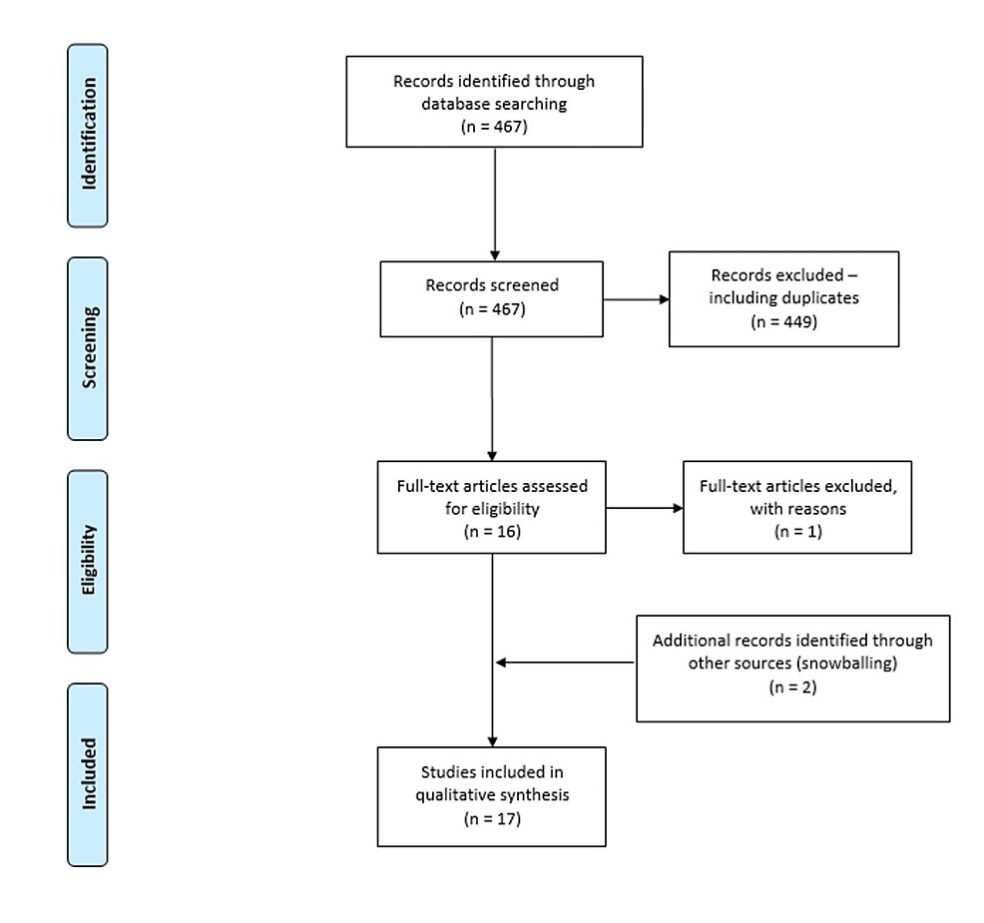
PRISMA flow diagram PRISMA: Preferred Reporting Items for Systematic Reviews and Meta-Analyses

Of the 467 studies assessed by title and abstract, 16 papers [3,8-22] were extracted and their full text was screened. One case report [22] was further excluded at this stage as it did not provide relevant data, such as MOI, management, or outcomes. Two more case reports [23,24] were included that were found in the reference list of our relevant studies. Seventeen case reports [3,8-21,23,24] were finally included (Figure 5).

Twenty-two cases (19 males, three females, Male:Female ratio 6.3:1), including our case, with a mean age of 33 years (range 4-70 years) were analyzed (Table 1).

**Table 1:** Summary of the ISAF cases Data include demographics, mechanism of injury, associated injuries, displacement status, scapula winging, initial and subsequent management, outcome, type of surgery, timing from diagnosis to treatment, and follow-up M: male; F: female; MVA: motor vehicle accident; N/A: non-applicable; ISAF: inferior scapula angle fractures

Author/year	Patient (age/ sex)	Mechanism of injury	Associated injuries	Displacement	Winging	Management (initial)/outcome	Management (subsequent)/outcome	Surgery performed	Timing from diagnosis to final treatment	Follow-up (months)
Peraino et al., 1977 [23]	57/M	Epileptic seizure	No	Unknown	Unknown	Conservative/successful			Immediate	No
Hayes and Zehr, 1981 [24]	25/M	Unknown	Cerebral contusion	Yes	Yes	Conservative/failed	Operative/successful	Surgical excision of the displaced bone fragment	10 months	12
Heyse-Moore and Sroker, 1982 [8]	13/F	Toboggan accident	Lateral body scapular fracture	No	Yes	Conservative/successful			23 days	4.5
	70/M	Fell on the street	Unknown	Unknown	Unknown	Conservative/successful			Immediate	2
Martin and Weiland, 1994 [9]	27/F	MVA	Flail chest, open tibia and fibula fractures, head injury	Yes	Yes	Conservative/failed	Operative/successful	Surgical excision of the displaced bone fragment	23 years (overlooked)	15
Brindle and Coen, 1998 [10]	17/M	Indirect trauma; extreme shoulder extension with elbow in extension	No	No	Yes	Conservative/successful			Immediate	6
Gupta et al., 1998 [11]	45/M	Pallet of bricks fell on the patient	Second lumbar spinal fracture	Yes	Yes	Operative/successful		ORIF with two-plate technique	7 months (overlooked)	24
Kaminsky and Pierce, 2002 [12]	16/M	Tackle during football game	No	Yes	No	Conservative/failed	Operative/successful	Surgical excision of the displaced bone fragment	3 months	4.5
Franco et al., 2004 [13]	47/M	Indirect trauma (prolonged cough)	No	No	Unknown	Conservative/failed	N/A (displaced fragment)		Immediate	3
Mansha et al., 2010 [14]	31/M	Fall - thrown out of car (army transportation)	No	Yes	Yes	Conservative/failed	Operative/successful	Suture repair of the avulsed fragment	2 years	3.5
Szopinski et al., 2012 [15]	5/M	Fall on rigid object	No	Yes	No	Operative/successful		Suture repair of the avulsed fragment	Immediate	3
Min et al., 2014 [16]	41/M	MVA	Unknown	Yes	Yes	Conservative/failed	Operative/successful	ORIF with 2-plate technique	12 months	24
	39/M	Fall from height	Unknown	Yes	Yes	Operative/successful		Suture repair of the avulsed fragment	Immediate	18
	55/M	Fall from height	Rib fractures	Yes	Yes	Operative/successful		Suture repair of the avulsed fragment	Immediate	12
Chang et al., 2016 [3]	43/M	Fall from height	No	Yes	Yes	Operative/successful		Suture repair of the avulsed fragment	1 month	3
	65/M	MVA	Multiple spinal fractures, head injury	Yes	Yes	Conservative/failed	N/A (lost to follow-up)		16 months	No
Speigner et al., 2016 [17]	51/M	Fall from stairs	No	Yes	Yes	Conservative/failed	Operative/successful	Bone anchor repair of the avulsed fragment	5 months (overlooked)	3
Park et al., 2017 [18]	10/M	MVA	Abdominal trauma, rib fractures, hemopneumothorax	Yes	Unknown	Operative/successful		Suture repair of the avulsed fragment	Immediate	24
Miller et al., 2018 [19]	4/M	Fall from stairs	No	No; deformation	No	Conservative/successful			Immediate	No
Ogawa et al., 2019 [20]	20/F	MVA	No	No	Yes	Conservative/failed	Conservative/successful		10 months (overlooked)	120
Edgington et al., 2020 [21]	12/M	Fall – sliding down waterslide	No	Yes	Yes	Conservative/successful			Immediate	12
Our case, 2021	42/M	Indirect trauma – extreme arm adduction	No	No	No	Conservative/successful			Immediate	3

There were 15 adult and seven pediatric cases (Adult:Pediatric ratio 2.1:1), with 18 years as the cutoff age for pediatric cases. The most common MOI was high energy injuries (77%, 17 cases); more specifically falls (41%, nine cases) [3,8,14-17,19,21], motor vehicle accident (MVA) - including toboggan accident (27%, six cases) [3,8,9,16,18,20], and direct trauma (9%, two cases) [11,12]. On the other hand, indirect trauma, such as extreme shoulder extension, prolonged cough, and extreme arm adduction (our case), was reported in 14% (three cases) [10,13]. Epileptic seizure was reported as MOI in one case (5%) [23], while one case had unknown MOI (5%) [24] (Table 2).

**Table 2:** Mechanism of injury MVA: motor vehicle accident; incl.: including

Mechanism of Injury	Cases (%)
Fall [3,8,14-17,19,21]	9 (41%)
MVA (incl. toboggan accident) [3,8,9,16,18,20]	6 (27%)
Indirect trauma [10,13]	3 (14%)
Direct trauma [11,12]	2 (9%)
Epileptic seizure [23]	1 (5%)
Unknown [24]	1 (5%)

Associated injuries were present in 32% (seven cases) [3,8,9,11,16,18,24], while 12 cases (55%) did not report associated injuries and in three cases (14%), it was unknown (Table 1). Scapula fractures, limb fractures, spinal fractures, rib fractures, thoracic injuries, abdominal injuries, and head injuries were reported.

Displacement of the ISAF fragment was found in 64% (14 cases) [3,9,11,12,14-18,21,24] (Table 1). Six cases (27%) did not report displacement [8,10,13,19,20], while in two cases (9%) it was unknown [8,23]. Winging of the scapula was found in 64% (14 cases) [3,8-11,14,16,17,20,21,24], while in 18% (four cases) it was not reported [12,15,19] and in another 18% (four cases), it was unknown [8,13,18,23]. In 32% (7 cases) there was a discrepancy between the displacement of the fracture and winging of the scapula; in 18% (four cases) there was no displacement, but winging was reported and in 14% (three cases), displacement was present but no winging was noted. Displacement was reported in 76% (13 cases) of the high-energy injuries, such as falls, MVAs, and direct trauma [3,9,11,12,14-18,21], whereas no displacement (100%, three cases) was reported in the indirect trauma cases [10,13].

**Figure 6:**
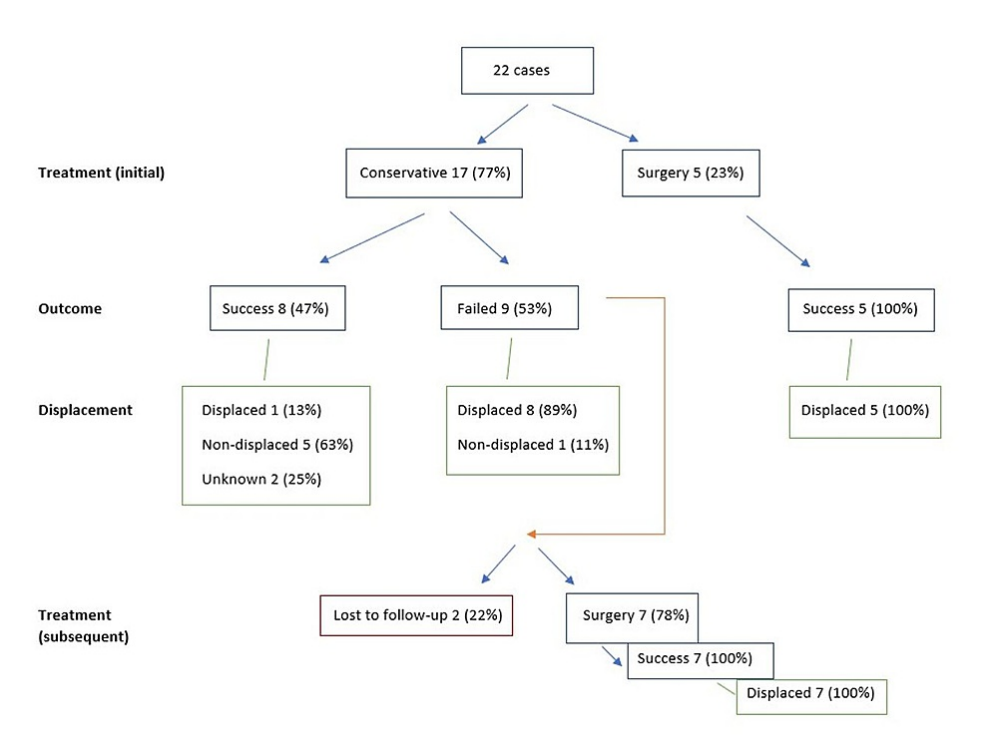
Management algorithm. ISAF tend to be treated initially conservatively; however, displacement strongly affects outcome. Surgical management (either early or after failed conservative treatment) yields 100% positive results and should be considered in displaced fractures. Conservative management should be considered in non-displaced fractures. ISAF: inferior scapula angle fractures

Initial management was conservative in 77% (17 cases) [3,8-10,12-14,16,17,19-21,23,24] and surgical in 23% (five cases) [11,15,16,18] (Table 1, Figure 6).

Of the cases treated conservatively, 47% (eight cases) [8,10,19-21,23] were successful, while in the other 53% (nine cases) [3,9,12-14,16,17,24] the treatment failed. Of the eight cases that were successful with conservative management, 63% (five cases) had no ISAF displacement [8,10,19,20], while 13% (one case) was displaced [21] and in two cases, displacement was unknown [8,23]. Of the nine cases that failed the conservative management, 89% (eight cases) were displaced [3,9,12,14,16,17,24], while 11% (one case) was not displaced [13]. For the conservatively treated failed cases, further management was explored. Seven cases (78%) underwent surgical treatment [3,9,12,14,16,17,24], while in other two cases, further management was unknown, or the cases were lost to follow-up [3,13]. Of the seven cases that were treated surgically after they failed conservative management, 100% (seven cases) had fracture displacement. Further to that, of the cases treated initially operatively [11,15,16,18], there was 100% success in the outcome (five cases) and of them, 100% (five cases) had ISAF displacement.

Overall, 12 cases underwent operative management; five cases were treated initially surgically [11,15,16,18], and another seven cases underwent subsequent surgical treatment after conservative management failure [3,9,12,14,16,17,24]. Four different surgical approaches were identified (Table 3).

**Table 3:** Type of surgery (as initial treatment or after failed conservative management) and displacement status of surgically managed ISAF ORIF: open reduction and internal fixation; ISAF: inferior scapula angle fractures

Type of Surgery	Cases	Initial treatment (cases)	Surgery after conservative failure (cases)	Displaced fractures (%)
Suture repair [3,14-16,18]	6	4	2	100%
Surgical excision of the displaced fragment [9,12,24]	3	-	3	100%
ORIF (2-plate technique) [11,16]	2	1	1	100%
Bone anchor repair [17]	1	-	1	100%

Suture repair was the most common procedure performed (50%, six cases) [3,14-16,18], followed by surgical excision of the ISAF fragment in 25% (three cases) [9,12,24]. Open reduction and internal fixation (ORIF) with the two-plate technique was also performed in 17% (two cases) [11,16], while bone anchor repair was performed in 8% (one case) [17]. Of the five cases that were treated surgically from the beginning, 80% (four cases) underwent suture repair [15,16,18], while 20% (one case) underwent ORIF with the two-plate technique [11]. Of the seven cases that were treated surgically after they failed the conservative management, 43% (three cases) underwent surgical excision of the ISAF fragment [9,12,24], while 29% (two cases) underwent suture repair [3,14]; ORIF with two-plate technique (one case) [16] and bone anchor repair (one case) were also reported [17]. Displacement of the ISAF fragment was noted in all cases that were treated operatively, either initially or after the failure of the conservative management.

Timing from ISAF diagnosis to the final treatment was reported in all cases (Table 1). It ranged from timely diagnosis and treatment to overlooked injuries that needed several months to treat. Immediate diagnosis and treatment were most reported in cases treated conservatively; however, one case of conservative treatment, which was successful, was overlooked for 10 months [20]. On the other hand, surgical management timing ranged from timely diagnosis and treatment to overlooked injuries that were treated after several months, with one case managed surgically 23 years after the injury [9]. Overall, 18% (four cases) were overlooked [9,11,17,20], three of them were managed operatively [9,11,17] and one conservatively [20].

Follow-up was reported in 86% (19 cases) [3,8-18,20,21,24] (Table 1). It ranged from no follow-up to 120 months (10 years), with a mean of 15.6 months. All authors rated the outcome as good/excellent using either only clinical and radiologic outcome or clinical and radiologic outcome and standardized outcome scores.

Discussion

ISAF are relatively rare; they most commonly occur in young adult males and the majority are considered high-energy injuries. Male and adult preponderance was noted; with six times more male and two times more adult cases compared to female and pediatric cases, respectively. Our findings are in agreement with Bartonicek et al., who also reported ISAF in adult males [25]; they have reported 20 adult and 11 pediatric ISAF (ratio 2:1) [25,26], similar to our review. ISAF fractures, however, have been reported by Bartonicek et al. as a relatively common scapula fracture in children (16% in pediatric scapula fracture series) [26], while rarely reported in adults (5% in adult scapula fracture series) [25]. Five different types of ISAF have been identified [25]; they can occur as single fractures or in combination with other fractures or injuries, according to their injury mechanism.

High-energy injuries comprise the majority of MOI for the reported cases. Falls, MVAs, direct trauma, and seizures have been reported in more than 85% of the cases. ISAF can also occur after indirect trauma (as also in our case) and are mostly avulsion fractures; those have been rarely reported with only three cases (including ours). However, as there are ISAF that were overlooked at initial work-up, we thus believe other ISAF can possibly exist that were either diagnosed late or never diagnosed.

Due to the variability of the inflicting injury mechanisms, no single presentation dominates. However, Ogawa et al. [20] reported that, as in any acute fracture, ISAF present with acute pain, local edema, tenderness, and pain or inability to perform full ROM of the injured shoulder. Winged scapula can also be present. The lower scapula is surrounded by several muscle attachments, such as serratus anterior, latissimus dorsi, and others. The serratus anterior muscle is innervated by the long thoracic nerve; injury to this nerve causes the classic winging of the scapula.

**Figure 7:**
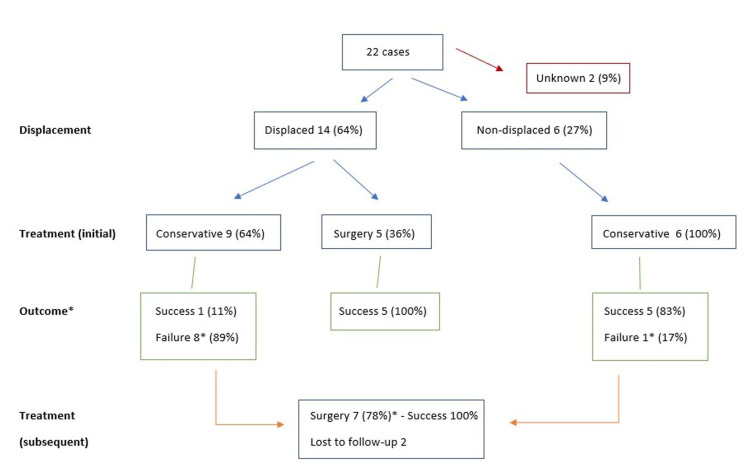
Management algorithm. Displaced fractures tend to fail conservative treatment and require surgical management, while non-displaced have good outcomes on conservative management * Of the nine cases that failed conservative treatment (eight displaced and one non-displaced fractures), 78% (seven cases) were treated surgically with 100% success; the other two cases were lost to follow-up.

Winging of the scapula, in most cases, can prognosticate fracture displacement; almost two-thirds of the patients had scapula winging and another two-thirds had ISAF displacement. Presence of displacement possibly affects both management and outcome. More specifically, all the non-displaced ISAF were treated conservatively, compared to the displaced fractures where two-thirds were treated conservatively and the other one-third underwent surgery (Figure 7).

The outcome of conservative management, however, was proportionate to the displacement status; almost 90% of the displaced fractures failed conservative treatment contrary to the non-displaced fractures where more than 80% were treated successfully conservatively. Furthermore, of the displaced fractures treated initially surgically, all reported treatment success. Displacement is also present in the majority of high energy trauma cases; when such MOI is encountered, caution should be taken in the management algorithm.

The treatment options reported were either conservative or surgical management (Figure 6). Tendency to treat ISAF initially conservatively was noted; more than 75% was treated initially conservatively, regardless of displacement status. More than half of the conservatively treated ISAF, however, failed initial management; of them, all but one fracture were displaced. Overall, surgical treatment yielded 100% positive outcome results, as both the initially and subsequently (after failed conservative management) surgically treated ISAF reported treatment success. On the contrary, of the cases that were treated conservatively, the majority were non-displaced fractures. We thus reach the same conclusion with the work by Chang et al. [3], that in case of displaced ISAF, surgical management should be the method of choice as it yields better outcome. Conservatively treated displaced fractures, otherwise, can develop painful non-union and possibly dysfunctional painful shoulder. Conservative management should be reserved for non-displaced ISAF, as it yields treatment success. In agreement with the above, in a recent ISAF series, the majority were treated conservatively; however, three ISAF were operatively managed and all fractures reported excellent/good outcome [26]. Edington et al. [21] is the only exception that successfully treated a displaced ISAF conservatively.

Suture repair of the ISAF was the most common type of surgery reported overall and the most common surgery performed as initial treatment. On the other hand, excision of the displaced fragment was the most reported surgery performed after failed initial conservative therapy, followed by suture repair. ORIF with two-plate technique and bone anchor was also reported. All surgery types, either as initial treatment or after failed conservative management, yielded positive outcomes; all surgeries were only performed in displaced ISAF. We thus believe that, in case of displaced fractures, where surgery is recommended as the treatment choice, suture (or bone anchor) repair should be considered first to preserve, with minimal intervention, the displaced fragment and restore the anatomy. However, if suture (or bone anchor) repair is not possible (i.e., in case of comminution) or it is the surgeon’s preference, ORIF or surgical excision can be considered.

Missed diagnosis and, therefore, treatment was also reported; of the missed ISAF, the majority were treated operatively and only Ogawa et al. [20] reported a conservatively treated overlooked ISAF after 10 months. We, therefore, believe that overlooked diagnosis should be approached operatively to avoid treatment failure. Further to that, although the majority of ISAF are single fractures (less than one-third of the cases report associated injuries), half of the overlooked cases had simultaneous more severe or life-threatening injuries. It is therefore important to maintain a high index of suspicion, especially in cases with concomitant associated injuries or more severe fractures, so that ISAF is not overlooked.

The outcome, either after successful conservative or surgical management, was reported as good/excellent; a wide range of follow-ups was reported. For surgically treated patients, a regular postoperative follow-up period should suffice. For conservatively managed ISAF, on the contrary, a minimum of three to six months of follow-up should be warranted, in our opinion, to avoid treatment failure (non-union, winging), especially in displaced ISAF, as noted by Edington et al. [21].

Our study has several limitations. The included studies comprised only case reports and are therefore of low quality. The term “displacement” is also not clearly defined for the ISAF. Furthermore, only a few studies reported standardized measurements to document improvement in certain functions. Another limitation is that the mode of treatment is not uniformly documented, and rehabilitation protocol is not reported in the studies. Finally, the number of the ISAF is small; therefore, solid conclusions and suggestions cannot be safely extracted and more cases are needed to implement our results.

## Conclusions

ISAF represent a rare entity that often presents simultaneous injuries elsewhere. Displacement status of the fracture should directly impact management algorithm, and therefore affects the outcome. Displaced fractures should be managed operatively, whereas in non-displaced fractures conservative treatment can be implemented. Adequate follow-up should be warranted to avoid treatment failure.
